# Discrepancies Between Explicit Feelings of Power and Implicit Power Motives Are Related to Anxiety in Women With Anorexia Nervosa

**DOI:** 10.3389/fpsyg.2020.618650

**Published:** 2021-02-09

**Authors:** Felicitas Weineck, Dana Schultchen, Freya Dunker, Gernot Hauke, Karin Lachenmeir, Andreas Schnebel, Matislava Karačić, Adrian Meule, Ulrich Voderholzer, Olga Pollatos

**Affiliations:** ^1^Department of Clinical and Health Psychology, Ulm University, Ulm, Germany; ^2^Treatment Center for Eating Disorders, Dritter Orden Hospital, Munich, Germany; ^3^ANAD e.V., Munich, Germany; ^4^Schön Klinik Roseneck, Prien am Chiemsee, Germany; ^5^Department of Psychiatry and Psychotherapy, University Hospital, Ludwig Maximilian University, Munich, Germany; ^6^Department of Psychiatry and Psychotherapy, University Hospital of Freiburg, Freiburg, Germany

**Keywords:** anorexia nervosa, power, implicit motives, anxiety, powerlessness, eating disorders

## Abstract

**Background:**

Several studies identified low subjective feelings of power in women with anorexia nervosa (AN). However, little is known about implicit power motives and the discrepancy between explicit feelings of power and implicit power motives in AN.

**Aim:**

The study investigated the discrepancy between explicit feelings of power and implicit power motives and its relationship to anxiety in patients with AN.

**Method:**

Fifty-three outpatients and inpatients with AN and 48 participants without AN were compared regarding subjective feelings of power and anxiety. Explicit power [investigated with the Personal Sense of Power Scale (trait focus) and a visual analog scale (state focus)], implicit power motives [investigated with the Multi-Motive Grid (MMG)] and trait anxiety [measured with the State-Trait Anxiety Inventory (STAI)], were assessed.

**Results:**

Explicit feelings of power (state and trait level) were lower in patients with AN compared to non-AN participants. No differences in implicit power motives were found when comparing the groups against each other. However, looking at the groups separately, women with AN had similar levels of implicit fear of losing power and hope for power, whereas woman without AN had significantly lower fear of losing power than hope for power. Focusing on discrepancies between powerful feelings and power motives, results were mixed, depending on the subscale of the MMG. Lastly, discrepancies between implicit power motives and explicit feelings of power were positively correlated with trait anxiety in AN patients.

**Conclusion:**

These findings underline that individuals with AN display significantly lower explicit feelings of power, however, they show similar implicit power motives compared to individuals without AN. The discrepancy between explicit feelings of power and implicit power motives is related to anxiety in AN and may represent a vulnerability factor to illness maintenance.

## Introduction

A *motive* has been defined as a predisposition to either approach particular incentives such as power, achievement or affiliation, or to avoid particular threats such as rejection, failure, or domination by others (Thrash et al., 2019). Previous research on human motivation has made a distinction between implicit- and explicit motives (Brunstein, 2018). Explicit motives refer to concrete self-assigned goals an individual strives for Brunstein et al. (1998). They are consciously and verbally expressed and can, therefore, be assessed with self-reports (Kollner and Schultheiss, 2014). In contrast, implicit motives describe spontaneously recalled and dispositional preferences for affective incentives that are assessed indirectly with picture-story exercises, such as the semi-projective Multi-Motive Grid (Sokolowski et al., 2000; Job et al., 2010). Thus, an individual’s explicit motives can be seen as consciously set goals while implicit motives refer to affect-driven motive dispositions that the individual is mostly unaware of (Schuler et al., 2019).

Implicit and explicit motives can diverge from one another (Schultheiss et al., 2009). This discrepancy between implicit- and explicit motives has been found to negatively impact an individual’s physical health and psychological wellbeing (Kehr, 2004; Schüler et al., 2008). For example, it has been linked to a decrease in volitional strength (Kehr, 2004) and life satisfaction (Hofer et al., 2006), as well as a higher rate of job burnout (Rawolle et al., 2016) and negative affect (Job et al., 2010). Baumann et al. (2005) consequently referred to the discrepancy between implicit- and explicit motives as a “hidden stressor” which affects the individuals’ health. The stressful impact of motive discrepancies can be explained as a consequence of a behavioral conflict: self-assigned goals are influenced by social demands and may not always align with unaware motives, leading to behavioral tendencies that diverge (Job et al., 2010). Consequently, motive discrepancy may represent a vulnerability factor to psychopathology (Hofer and Chasiotis, 2003; Brandstatter et al., 2016). Despite this assumption, the impact of motive incongruence has rarely been studied in clinical samples.

The few studies that have focused on this issue in a clinical context have highlighted that motive incongruence may play a crucial role in symptom generation and maintenance (Langan-Fox et al., 2009; Langan-Fox and Canty, 2010). Regarding anorexia nervosa (AN), for example, Frank et al. (2019) proposed a model linking motive discrepancy to anxiety, which in turn affects negative eating behavior. The conscious goal to restrict food intake and to lose weight in AN patients conflicts with the implicit basic instinct to gain weight for survival, causing an internal conflict and, consequently, anxiety (Frank et al., 2019). This, in turn, might reinforce food restrictive behavior in order to avoid losing control (Frank et al., 2019). A similar pattern has been observed in non-clinical samples. For example, Job et al. (2010) found that motive incongruence was associated with unhealthy eating behavior in college students, mediated by negative affect. Based on these findings, continuing the investigation of implicit motives in AN patients seems crucial to gain a deeper understanding of potential internal conflicts that may generate and maintain anxiety and disordered eating. Anxiety is a particularly important outcome variable to investigate, as it represents one of the key comorbidities in AN, with studies reporting rates as high as 56% (Blinder et al., 2006). It also represents a factor that enhances the severity, chronicity and treatment resistance of the eating disorder (Kaye et al., 2004) and can persist after recovery from AN (Federici and Kaplan, 2008).

One motive that may be particularly relevant in the context of eating disorders is the *power* motive. Several studies have shown that women with AN display lower subjective (i.e., explicit) feelings of power than women without AN (Wolff and Serpell, 1998; Schwitzer et al., 2001; Troop et al., 2003; Woolrich et al., 2008). They also assign themselves a low social rank and tend to feel inferior to others (Troop et al., 2003; Bellew et al., 2006). A perceived low social rank and feelings of inferiority are associated with negative affect, such as shame (Ferreira et al., 2015), self-criticism (Pinto-Gouveia et al., 2014), and anxiety (Bellew et al., 2006). These, in turn, may further contribute to feelings of perceived powerlessness (Woolrich et al., 2006).

In this context, it is important to highlight that perceived power seems to be highly relevant regarding food consumption and disordered eating. For example, Guinote (2010) found that hunger predicted food intake in powerful but not in powerless individuals and powerful individuals consumed more appetizing- and less non-appetizing food compared to powerless individuals. Furthermore, Kunstman et al. (2014) found that experiencing power could increase caloric intake in participants with AN symptoms and high self-oriented perfectionism.

Despite strong evidence for low subjective feelings of power in women with AN and its proposed link to pathology, little is known about *implicit* power motives in this clinical cohort. It is, for example, uncertain whether women with AN have a higher, lower, or similar implicit power motive compared to women without AN. However, it is likely that they have a similar implicit power motive to women without AN as previous research has suggested that the eating disorder serves an attempt to defeat feelings of powerlessness (Bruch, 1979; Wolff and Serpell, 1998), which could be reflected in a high implicit power motive.

Furthermore, it is noteworthy that research on powerlessness in AN has mainly relied on qualitative data (Woolrich et al., 2006; Duncan et al., 2015) and has not integrated validated quantitative measurements that focus specifically on power, such as the Personal Sense of Power Scale by Anderson et al. (2012) or the Multi Motive Grid power-subscale by Sokolowski et al. (2000). In this study, we would like to breach this gap in the literature by examining whether there is a discrepancy between implicit power motives and explicit feelings of power in AN and by investigating how a possible discrepancy between these two variables might relate to feelings of anxiety. Based on existing theoretical models and previous research, we propose four hypotheses:

1. Women with AN display significantly lower explicit feelings of power on the state and the trait level than women without AN.
2. Women with AN have similar implicit power motives as women without AN.
3. Women with AN show a higher discrepancy between explicit feelings of power (trait level) and implicit power motives than women without AN.
4. Discrepancies between implicit power motives and explicit feelings of power are positively correlated with anxiety in AN patients.

## Materials and Methods

### Sample Characteristics

Fifty-three female patients with AN were recruited in two different outpatient clinics for eating disorders in Munich, Germany [ANAD e.V. and the Treatment Centre for Eating Disorders (TCE) at Dritter Orden Hospital] as well as the inpatient ward of Schoen Clinic Roseneck in Rosenheim, Germany. All patients had to be diagnosed with AN as defined by the DSM-5 criteria, determined by a semi-structured interview [SKID-5 cv (Beesdo-Baum et al., 2019)] by a clinical psychologist. Another inclusion criterion was that participants had to be over 18 years old. Patients with AN had a mean age of 24.70 years (*SD* = 7.12) and a mean body mass index (BMI) of 15.51 kg/m^2^ (*SD* = 2.09). 42 patients were diagnosed with restrictive AN, nine with binge/purging AN, and two with atypical AN.

The control sample consisted of 48 women without AN, recruited via advertisement at Ulm University. In the control group, mean age was 22.58 years (*SD* = 4.12), and mean BMI was 21.89 kg/m^2^ (*SD* = 2.73). Inclusion criteria were (1) no present psychiatric or somatic disorder and age over 18 years. Groups did not differ in age [*t*(84.824) = -1.848; *p* = 0.068] but, expectedly, differed in BMI [*t*(99) = 13.089; *p* < 0.001]. Further demographic information such as trait anxiety scores, illness duration, number of previous hospital admissions and scores of the Eating Disorder Inventory-2 are presented in Table 1.

**TABLE 1:** Descriptive statistics.

	**Control group**		**Anorexia nervosa**		**Test statistics**		
Variable	**M**	***SD***	**M**	***SD***	***T***	**df**	***p***
Age	22.58	4.12	24.70	7.12	−1.848	84.824	0.068
BMI	21.80	2.73	15.51	2.09	13.089	99	0.000
STAI trait	40.69	7.82	60.23	10.86	−10.279	99	0.000
Length of eating disorder	–	–	9.03	8.23	–	–	–
No. hospital admissions	–	–	3.59	3.07	–	–	–
EDI 2	–	–	314.19	63.39	–	–	–

### Measures

#### Subjective Feelings of Power

##### Personal Sense of Power Scale (SOPS)

To assess the explicit trait power of participants, the Personal Sense of Power Scale by Anderson et al. (2012) was used. It comprises eight items on a six-point scale, ranging from strongly disagree (=1) to definitely agree (=6). In our study, the McDonalds ω was excellent (ω = 0.909) indicating that the SOPS is as an internally reliable measurement (Hayes and Coutts, 2020). An example of a statement was “I think I have a great deal of power.” Some of the statements were reverse-scored to prevent response bias.

##### Visual *Analog Scale* (VAS) (state level)

To investigate participants’ explicit state power, they had to indicate how powerful they felt at the present moment on a visual analog scale. They had to mark their answer with a pen on a line, ranging from *no power at all* (=0) to *high power* (=100). The VAS for the power scales was embedded between a scale assessing current energy level and strength to avoid priming the participants with the focus of the study.

##### *Multi-Motive Grid (MMG*)

The MMG by Sokolowski et al. (2000) was used to assess implicit power. This semi-projective assessment aims to evaluate an individuals’ implicit motivation for affiliation, achievement, and power. Each motive has two dimensions. For example, regarding the power motive, the MMG distinguishes between *hope for* power (HP) and *fear of losing* power (FP). The HP dimension primarily represents an individuals predisposition to influence other people or to gain power and status over others (Schmalt et al., 2010). On the other hand, the FP dimension is concerned with avoiding the loss of standing and the fear of being overpowered by other people (Schmalt et al., 2010). Fourteen drawings with different ambivalent social situations were presented. Below the picture, a range of statements was shown referring to each motive and its dimension. Participants were instructed to judge whether the presented statement fitted the given situation or not, by circling “yes” or “no.” Examples of power statements were “anticipating to lose standing” or “hoping to acquire a good standing.” For each picture, a single motive score was calculated to obtain a global score for each of the six motive components. Scores ranged from zero to twelve. For our study, only the power scores were used. The MMG has demonstrated good internal consistency and reliability in previous research (Sokolowski et al., 2000; Kehr, 2004). In our study, both MMG power subscales showed acceptable reliability (MMG HP: α = 0.762; MMG FP. α = 0.712).

#### Anxiety Level

The State-Trait Anxiety Inventory (Laux et al., 1981) is a widely used and validated questionnaire for the assessment of anxiety symptoms. For this study, only the trait anxiety scale was used to assess individuals’ dispositional anxiety level. The reason, therefore, was that the MMG and the Personal Sense of Power Scale (that were used to calculate power motive discrepancies) both also focus on trait-like constructs rather than situational feelings of power. The trait anxiety scale consists of 20-items and a four-point scale ranging from *almost never* (=1) to *almost always* (=4). Sum scores can range from 20 to 80, with higher scores indicating higher anxiety. In our study the McDonalds ω was excellent (ω = 0.920) indicating that the trait scale was as an internally reliable measurement.

### Procedure

The study was conducted in accordance with the Declaration of Helsinki and ethical approval was obtained from the Institutional Review Board of Ulm University (Protocol Nr 109/15). In a first step, participants were informed about the study procedure and signed an informed consent to take part. The AN patient sample was assessed in a separate, quiet therapy room of the outpatient clinics ANAD e.V., and TCE in Munich, as well as Schoen Clinic Roseneck in Rosenheim. The non-AN sample was tested in the laboratories of the Clinical- and Health Psychology Department of Ulm University. The participants completed the different questionnaires in their own time with the experimenter present. At the end of the study, participants received either course credit or monetary compensation.

### Statistical Analyses

Data analyses were performed using the program IBM SPSS Statistics 26 (SPSS, Chicago). Group differences (AN vs. non-AN) in implicit and explicit power variables (hypothesis 1–3) were investigated using independent samples *t*-tests. Homogeneity of variance was tested using Levene’s Test. When the assumption of homogeneity was violated, Welch’s *t*-test was used. Regarding hypothesis 3, MMG scores and SOPS scores were *z*-standardized and then absolute differences between the *z*-scores were calculated (Rawolle et al., 2016). Pearson correlations were conducted for the different power measures and anxiety separated by the groups (hypothesis 4). *P*-values less than 0.05 defined significant results. Two-sided tests were used for all hypotheses.

## Results

**FIGURE 1:**
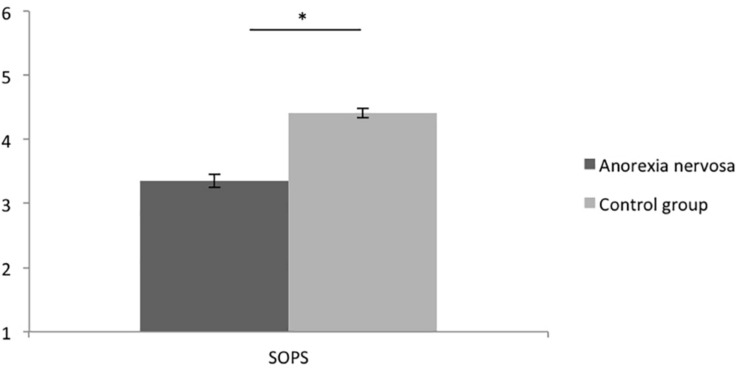
Means of the Personal Sense of Power Scale (SOPS) of women with and without AN. Error bars represent SE.

**FIGURE 2:**
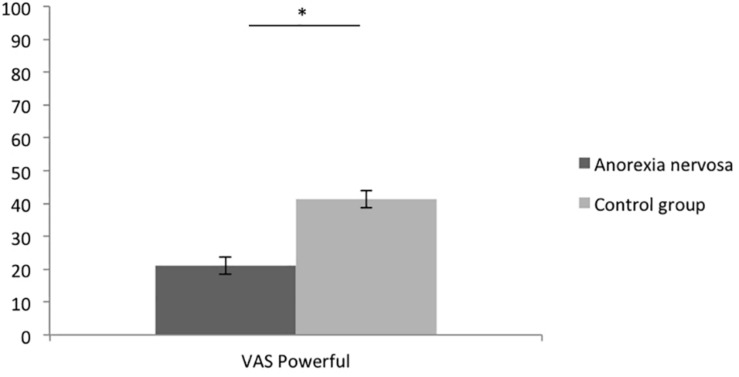
Means of the Visual Analog Scale of Power (VAS) of women with and without AN. Error bars represent SE.

Confirming hypothesis 1, women with AN (*M* = 3.35; *SD* = 0.78) showed significantly lower Personal Sense of Power (SOPS) values than women without AN (*M* = 4.40, *SD* = 0.49), [*t*(88.888) = 8.136, *p* < 0.001, *d* = 1.726, see Figure 1. Regarding the VAS power scores, women with AN also displayed significantly lower scores (*M* = 21.08, *SD* = 20.64) than women without AN (*M* = 41.38, *SD* = 21.55), *t*(97) = 4.786, *p* < 0.001, *d* = 0.972 (see Figure 2). Thus, women with AN showed significantly lower explicit feelings or power than women without AN (on the state and the trait level).

**FIGURE 3:**
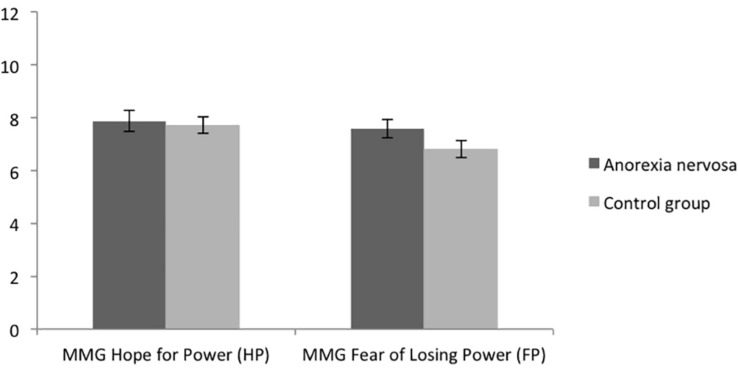
Means of the Multi-Motive Grid (MMG) fear of losing power and hope for power dimension of women with and without AN. Error bars represent SE.

As expected, there was no significant difference between groups regarding the MMG hope for power dimension, *t*(99) = -0.289, *p* = 0.773, *d* = 0.058, (see Table 2 and Figure 3) and no significant difference between groups regarding the MMG fear of losing power dimension, *t*(99) = -1.472, *p* = 0.144, *d* = 0.296. Thus, the implicit power motives did not differ between groups (hypothesis 2).

**TABLE 2:** Means and standard deviations of power measurements and MMG/personal sense of power scale discrepancies.

	**Control group**		**Anorexia nervosa**		**Test statistics**		
Variable	**M**	***SD***	**M**	***SD***	***T***	**df**	***p***
SOPS	4.40	0.49	3.35	0.78	8.136	88.888	0.000
VAS powerful	41.38	21.55	21.08	20.64	4.786	97	0.000
MMG hope for power (HP)	7.71	2.69	7.87	2.85	−0.289	99	0.773
MMG fear of losing power (FP)	6.81	2.42	7.58	2.81	−1.472	99	0.144
MMG HP/SOPS discrepancy	0.92	0.74	1.07	0.74	−1.027	99	0.307
MMG FP/SOPS discrepancy	1.05	0.78	1.43	0.97	−2.157	99	0.033

Regarding hypothesis 3, women with AN (*M* = 1.43, *SD* = 0.97) displayed significantly higher discrepancies between the MMG fear of losing power dimension and the SOPS than women without AN (*M* = 1.05, *SD* = 0.78), *t*(99) = -2.157, *p* = 0.033, *d* = 0.434. No differences between the groups regarding discrepancies between the MMG hope for power dimension and the SOPS were found, *t*(99) = -1.027, *p* = 0.307, *d* = 0.206) (please refer to Table 2 and Figure 2). Thus, this hypothesis was partially supported. Further analysis revealed that there was no significant difference between the MMG hope for power dimension and the MMG fear of losing power dimension in the AN-group (Table 3). However, in the non-AN group, the MMG fear of losing power dimension was significantly lower than the MMG hope for power dimension (Table 3).

**TABLE 3:** Differences between MMG HP and MMG FP.

**Group**	**Variables**	***N***	**M**	***SD***	***T***	**df**	***p***
Control group	MMG hope for power (HP)	48	7.71	2.69	2.170	47	0.035
	MMG fear of losing power (FP)	48	6.81	2.42			
Anorexia nervosa	MMG hope for power (HP)	53	7.87	2.85	0.534	52	0.595
	MMG fear of losing power (FP)	53	7.58	2.81			

**FIGURE 4:**
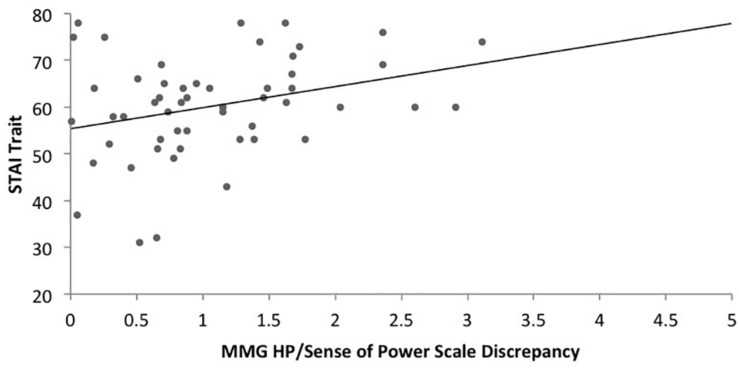
Scatterplot of the correlation between the Hope for Power (MMG)/Personal Sense of Power Scale discrepancy and trait anxiety in women with AN.

**FIGURE 5:**
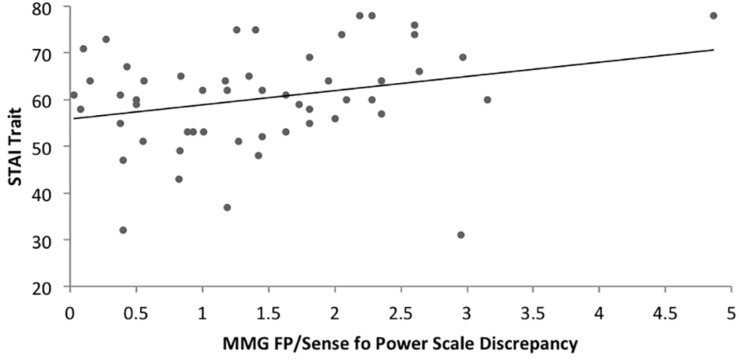
Scatterplot of the correlation between the Fear of Losing Power (MMG)/Personal Sense of Power Scale discrepancy and trait anxiety in women with AN.

Regarding hypothesis 4, there was a significant positive correlation between the MMG FP/SOPS discrepancy and trait anxiety (*r* = 0.271, *p* = 0.050) (Figure 4) as well as a significant positive correlation between the MMG HP/SOPS discrepancy and trait anxiety (*r* = 0.307, *p* = 0.025) for the anorexia nervosa group (Figure 5). No significant correlations were found for the control group regarding the MMG FP/SOPS discrepancy and trait anxiety (*r* = -0.229, *p* = 0.118) and the MMG HP/SOPS discrepancy and trait anxiety (*r* = 0.044, *p* = 0.767).

Exploratory analysis also revealed a significant positive correlation between the MMG FP/SOPS discrepancy and the EDI-2 in the AN group, (*r* = 0.29, *p* = 0.035). No significant positive correlation between the MMG HP/SOPS discrepancy and the EDI-2 was found (*r* = 0.184, *p* = 0.186). Regarding the MMG FP variable on its own, it significantly correlated with the EDI-2 in the AN group (*r* = 0.372, *p* = 0.006).

## Discussion

The study aimed to evaluate the discrepancy between implicit power motives and explicit feelings of power and its relationship with anxiety in women with- and without AN. As expected, the AN sample displayed significantly lower explicit power (on the trait- and state level) than the non-AN sample. In comparison, no differences between implicit power were found when comparing the groups against each other. However, looking at the groups separately, women with AN had similar levels of implicit fear of losing power and hope for power, whereas woman without AN had significantly lower fear of losing power than hope for power. Regarding the explicit- and implicit power discrepancies, results were mixed. Whereas there was a higher discrepancy between the Personal Sense of Power Scale and the MMG fear of losing power dimension in patients with AN, no significant differences were found regarding the discrepancy between the Personal Sense of Power Scale and the MMG hope for power dimension. Lastly, higher discrepancies between implicit power motives and explicit feelings of power were associated with higher levels of anxiety in patients with AN. No correlations were found regarding the non-AN sample.

The finding that individuals with AN displayed lower explicit feelings of power is in keeping with previous research findings highlighting that women with AN report feelings of powerlessness during their illness (Schwitzer et al., 2001; Troop et al., 2003; Woolrich et al., 2006; Duncan et al., 2015). It also fits in with theoretical models that highlight the presence of powerlessness as a key factor of illness pathology (Bruch, 1979; Wolff and Serpell, 1998). To our knowledge, this is the first study that employed the Personal Sense of Power Scale (Anderson et al., 2012) in AN patients. As it showed good internal consistency, it could be valuable to integrate as a diagnostic tool during clinical treatment, to assess the individuals’ explicit power level before and after interventions.

Furthermore, our finding showed that no differences between both groups were found for the implicit power motives, indicating that women with AN have similar predisposition to approach power and fear of losing power, as women without AN. Although no previous study used the MMG in an AN sample, our finding is congruent with studies that employed the MMG in other clinical samples, such as individuals with remitted major depression- and bipolar disorder (Fuhr et al., 2014) that also found no difference between individuals with and without psychopathology.

Regarding discrepancies between implicit power motives and explicit feelings of power in AN, the results were mixed. The finding that women with AN showed a significantly higher discrepancy between their explicit feelings of power and the fear of losing power dimension of the MMG, compared to women without AN, is in keeping with our expectation. This finding is concerning, as discrepancies in implicit- and explicit motives have been linked to psychopathology and stress (Baumann et al., 2005; Job et al., 2010; Rawolle et al., 2016). As the fear of losing power dimension was also significantly positively correlated with trait anxiety and eating pathology (EDI-2) in the AN group, decreasing the fear of losing standing and being overpowered by others could represent an important therapy goal.

Considering the findings further, individuals with AN did not show a significantly higher discrepancy between explicit feelings of power and the hope for power dimension of the MMG than women without AN. In this context, we found that the hope for power was significantly higher than the fear of losing power in the non-AN group. On the other hand, implicit approach tendencies toward power and the fear of losing power were similar in the AN group. Having similarly high levels of fear of losing power and hope for power has been described as a vulnerability factor for an approach-avoidance conflict (Schmalt et al., 2010). Therefore, future research should put particular emphasis on ways in which similar levels of approaching power and avoiding the loss of power can relate to eating pathology and disadvantageous behavior in individuals with AN.

In keeping with our prediction, the discrepancy between implicit power motives and explicit feelings of power was linked to trait anxiety, only in the AN group. This finding is in line with previous research highlighting that the discrepancy between implicit- and explicit motives is associated with anxiety in women with AN (Frank et al., 2019). This observation further underlines the importance of developing strategies to reduce the discrepancy between implicit- and explicit power motives in AN. However, little is known about how this can be achieved (Job, 2007). As the explicit power motive in AN is significantly reduced compared to non-AN women, it would be beneficial to firstly increase subjective feelings of power in women with AN. This idea is congruent with a systematic review meta-synthesis of qualitative research by Duncan et al. (2015) that identified regaining subjective feelings of power as crucial for illness recovery. One promising approach could be to practice power posing with individuals with AN. Power posing refers to the adoption of an expansive bodily posture (Carney et al., 2010, 2015). It is a non-verbal, body-focused technique that has repetitively been found to increase individuals’ feelings of power (Cuddy et al., 2018). It may be particularly helpful as recent studies in the field of eating disorders highlighted beneficial effects integrating body-centered interventions into treatment (Artoni et al., 2020).

To our knowledge, this is the first study investigating discrepancies between explicit feelings of power and implicit power motives and their relationship with anxiety in AN patients. However, our research needs to be interpreted, considering some limitations. Firstly, our AN sample was heterogeneous. For example, the participants differed regarding the AN subtype, comorbidities (e.g., depression and anxiety), as well as stages of recovery (in- and outpatients). Therefore, the study could be replicated and refined differentiating between the subtypes of AN and comparing participants during the acute illness and after recovery. Secondly, these data are cross-sectional. They should be extended by experimental and longitudinal data such as an ecological assessment to understand how subjective feelings of power and/or power motives fluctuate (e.g., throughout the day, with food intake as well as over the course of standardized treatment).

Furthermore, the investigation of the implicit power motive was based on the Multi Motive Grid by Sokolowski et al. (2000). Other tests that investigate implicit motives include the Picture Story Exercise (PSE) (McClelland et al., 1989), the Operant Motive Test (OMT) (Kuhl and Scheffer, 1999) or the Pictorial Attitude Implicit Association Test (PA-IAT) (Slabbinck et al., 2011). Thus, it could be valuable to replicate the study using an alternative implicit motive assessment. In this context, we mainly focused on the power dimension. Consequently, it would be interesting to investigate how individuals with and without AN differed regarding their implicit achievement- or affiliation motives.

Regarding clinical implications, our study has highlighted that low explicit feelings of power (on the state- and trait level) are present in AN patients. They should be explored in therapy, as they could represent a vulnerability factor regarding disordered eating and illness maintenance. In a therapeutic context, one could for example track explicit feelings of power (state level) using the visual analog scale at different points during the day or during episodes of disordered eating (e.g., before food intake, after food intake, during starvation, after purging etc.). Doing this could underline the connection between the individuals’ incentive to obtain power and avoid feelings of powerlessness through disordered eating. As our exploratory analysis also revealed a significant positive correlation between the MMG FP/SOPS discrepancy and the EDI-2, future studies should put a particular focus on how motive incongruence could be linked to eating pathology. Furthermore, one could assess individuals’ discrepancies between explicit feelings of power and power motives before, during and after treatment, to get a better understanding of possible inner conflicts and whether therapy is successful in reducing this incongruence.

## Conclusion

To sum up, our data provide evidence that explicit feelings of power are lower in women with AN than women without AN. However, there seems to be no significant difference regarding implicit power motives when comparing the groups against each other. Looking at the groups separately, women without AN display a significantly lower implicit fear of losing power than hope for power. In women with AN, the fear of losing power is similarly high as the hope for power. Moreover, the discrepancy between explicit feelings of power and implicit power motives is positively associated with anxiety in AN. It seems worthwhile to focus on the assessment of power in AN patients and to develop interventions that can address feelings of powerlessness.

## Data Availability Statement

The data that support the findings of this study are available from the corresponding author, (FW), upon reasonable request.

## Ethics Statement

The studies involving human participants were reviewed and approved by the Institutional Review Board of Ulm University. The patients/participants provided their written informed consent to participate in this study.

## Author Contributions

FW, DS, GH, KL, AS, and OP conceptualized the research idea and planned the experiments. FW carried out the experiments. FW, DS, and AM contributed to data analysis. FW, DS, GH, AM, UV, and OP contributed to the interpretation of the results. FW, DS, and FD wrote the manuscript. All authors provided critical feedback and helped shape manuscript.

## Conflict of Interest

UV, AM, and MK were employed by Schön Klinik. The remaining authors declare that the research was conducted in the absence of any commercial or financial relationships that could be construed as a potential conflict of interest.
